# Assessing consumers’ perception and demand on the community pharmacists’ dispensing

**DOI:** 10.1186/s40545-023-00609-1

**Published:** 2023-11-29

**Authors:** Yapp Wen Xuan, Hui Poh Goh, Inayat Ur Rehman, Naeem Shafqat, Yaser Mohammed Al-Worafi, Long Chiau Ming, Andi Hermansyah

**Affiliations:** 1 PAP Rashidah Sa’adatul Bolkiah Institute of Health Sciences, Universiti Brunei Darussalam, Gadong, BE1410 Brunei; 2 Department of Pharmacy, Abdul Wali Khan University Mardan, Mardan, 23200 Pakistan; 3 College of Medical Sciences, Azal University for Human Development, Sana’a, Yemen; 4 College of Pharmacy, University of Science and Technology of Fujairah, P.O. Box 2202, Fujairah, United Arab Emirates; 5 Department of Pharmacy Practice, Faculty of Pharmacy, Universitas Airlangga, Surabaya, 60115 Indonesia; 6 School of Medical and Life Sciences, Sunway University, 47500 Bandar Sunway, Selangor Malaysia

**Keywords:** Consumers’ perception, Affordability, Health policy, Dispensing separation system, Medicine, Medicine access

## Abstract

**Background:**

This study aimed to assess the general public's perception of services provided by community pharmacies, their willingness to utilize these services, their satisfaction with and understanding of community pharmacists, and their views on dispensing separation and pharmacy medicines (P medicines).

**Methods:**

An online cross-sectional study was conducted, in which questionnaires were distributed among the general public. A novel questionnaire was designed and validated specifically for this study. It was composed of six sections: demographics, pharmacy usage and service preferences, understanding and satisfaction with pharmacists, views on dispensing separation, private community pharmacies, and knowledge of P medicines. Statistical analyses such as one-way ANOVA, independent *t* test, and binary logistic regression were employed, with a *p* value of < 0.05 considered statistically significant.

**Results:**

The study received 222 responses. The majority of the respondents were females within the 20–29-year-old age group (62.2%). Most respondents preferred to consult doctors for medical treatment, with their primary reason for visiting community pharmacies being to collect prescribed medicines. About 52.7% of respondents expressed their willingness to avail of screening services and treatment for minor illnesses at community pharmacies. A statistically significant difference was found among different age groups regarding their views on the dispensing separation system, with those aged 41–50 years demonstrating higher scores. However, the binary logistic regression analysis did not reveal any statistical significance when comparing the understanding of P medicines among respondents.

**Conclusions:**

In general, the public prefers to consult doctors for medical treatment and visit community pharmacies predominantly to collect prescriptions or purchase over-the-counter medications. Nonetheless, they are also open to utilizing services provided by community pharmacists, particularly screening services and treatment for minor illnesses.

## Background

Pharmacy is one of the largest healthcare professional groups worldwide [1]. In the past, the pharmacist’s role involved obtaining chemical ingredients, manufacturing, and supplying medicinal products—a role now performed by the pharmaceutical industry. Today, the primary roles of community pharmacists are dispensing and retailing [2]. Despite their extensive professional training, the capabilities of community pharmacists are underutilized. Their roles should ideally be expanded to more clinically oriented, patient-centered services. Such an expansion could enhance health outcomes by promoting better medication adherence, reducing drug-related adverse events, and decreasing unnecessary provider visits, hospitalizations, and readmissions [1]. Furthermore, growing knowledge and technological advancements have heightened expectations for pharmacists to provide more efficient primary care services [3].

There are two main types of pharmacies: hospital and community pharmacies. Hospital pharmacies are located within hospitals, while community pharmacies, typically situated within the community, are more accessible [4]. In some countries, the public prefers visiting community pharmacies due to their convenience—no appointments are required, and they offer extended opening hours [5]. These pharmacies are often chosen as the first point of contact for minor illnesses, such as coughs and colds [5]. Unlike most countries where community pharmacies are privately owned, in Brunei, both health center and private pharmacies are categorized as community pharmacies.

According to the Ministry of Health (MOH), the community pharmacy services in Brunei are confined to dispensing medications as prescribed by doctors, adhering to Good Dispensing Practice (GDP), providing patient counseling about their medications, and supplying floor stock medications to peripheral clinics [6]. These services constitute core pharmacy services rather than the enhanced or expanded community pharmacy services for patient-centered care [7].

Systematic reviews and meta-analyses have demonstrated that the expansion of clinical services by community pharmacies has been successfully adopted and implemented [8, 9]. Recently, New Zealand implemented a substantial reform of its community pharmacy sector through the new Community Pharmacy Services Agreement (CPSA). The country transitioned from a reimbursement-per-dispensing model to a patient-centered services model [2]. To execute the clinical activities, consultation rooms and additional staff with appropriate expertise may be required. Consumer demand is also crucial for successful implementation [10].

Many countries already offer enhanced services. For instance, the UK introduced expanded services in 2005 [11]. The services are divided into three categories: essential, advanced, and locally commissioned. Essential services, such as dispensing and signposting to patients, are traditional services originally provided by community pharmacies. Advanced services require more patient interaction and communication, with pharmacies offering one-to-one consultations and medication reviews. As a result, consultation rooms have become more common in community pharmacies to ensure privacy. Finally, locally commissioned services are broader and cover a wide range of medication and public health services, such as managing minor illnesses (e.g., cough and cold), offering lifestyle advice, and providing sexual health services [12]. A published study revealed that most participants are willing to accept the majority of these extended services [13]. However, patients may only be familiar with the traditional roles of community pharmacists, namely, checking prescriptions, dispensing, and counseling on medication, as indicated by studies assessing the public's views on community pharmacies and pharmacists [14, 15].

Pharmacists’ dispensing roles can be constrained by the classification of medicines, which are categorized into three types: Prescription Only Medicines (POM), Pharmacy Medicine (P medicines), and General Sales List (GSL). POM can only be prescribed by doctors and dispensed with a valid and complete prescription. P medicines, on the other hand, can be purchased from a pharmacy without a prescription but require the presence of a pharmacist. These medicines are usually stored behind the counter in pharmacies and not in open areas. GSL medicines can be sold anywhere—retail stores, supermarkets, or other shops [16]. In Brunei, the Brunei Darussalam Medicines Control Authority (BDMCA) regulates medication classification.

The Poisons List, overseen by the Poisons Act, regulates the importation and sales of poisons, including prohibitions and provisions related to poison sales [17]. The current Poison List allows only a limited number of items to be dispensed by pharmacists without a prescription. This limitation, along with an imbalance in classified medications and a lack of dispensing separation, has restricted the roles of pharmacists in managing minor illnesses, such as mild pain and cough [17].

Information on the expanded or enhanced community pharmacy services in Brunei Darussalam remains scarce, hindering the assessment process needed for successful implementation of these services. Thus, this study aims to provide a clearer understanding of the general public’s perception of services offered by community pharmacies and their willingness to utilize them. It also seeks to evaluate public satisfaction with and understanding of community pharmacists. Finally, the study will examine attitudes and behaviors concerning access to P medicines and dispensing separation. The data gathered from this study should reflect Brunei’s current situation more accurately and may serve as a valuable reference for future policy development and implementation.

## Methods

### Study design, site, and participants

This online cross-sectional survey was conducted from March 2023 to April 2023 among the general public of Brunei Darussalam. Questionnaires were distributed at the RIPAS hospital and community pharmacies in accordance with the Ministry of Health in Brunei. Distribution also occurred via social media platforms (WhatsApp and Instagram), and emails. Posters containing relevant information and a QR code linking to the questionnaire (English version) were displayed at RIPAS hospital and community health centers. Emails were sent to university students via their respective registrars or departments. The targeted universities and colleges included Universiti Brunei Darussalam (UBD), Universiti Teknologi Brunei (UTB), Universiti Islam Sultan Sharif Ali (UNISSA), Institute of Brunei Technical Education (IBTE), Laksamana College of Business (LCB), and Politeknik Brunei (PB). The poster and QR codes were also included in emails, WhatsApp messages, and Instagram stories.

The inclusion criteria for this study were: (i) individuals aged 18 years and above and (ii) individuals residing in Brunei Darussalam. The exclusion criteria included: (i) adults temporarily residing in Brunei Darussalam and (ii) adults unwilling to participate.

### Sample size

This study used a convenience sampling strategy. In this non-probability sampling approach, study inclusion primarily depended on potential participants’ convenience and willingness. This method does not require a predetermined sample size nor aims to be fully representative of the population. The objective was to expedite data collection and glean insights from an easily accessible group of individuals exhibiting characteristics relevant to the research question.

### Study instrument

A questionnaire was designed after conducting a literature review of relevant studies [4, 13, 15, 18], to align with the local context in English (see Appendix 1). The Delphi technique was employed to reach a consensus on group opinion. To facilitate the Delphi method, the panel consisted of the principal investigator of this study along with experts specialized in the area of pharmacy and practice. The questionnaire comprises 44 questions divided into six sections. The sections gather information on respondents’ demographics, pharmacy usage, service preferences, understanding and satisfaction with pharmacists, views on the dispensing separation system, perception of private community pharmacies, and knowledge of P medicines. The questions were a mix of multiple-choice, checkbox ticking, Likert scale, and short-answer questions. Likert scale question scores ranged from 1 (very satisfied/very good) to 5 (very unsatisfied/very bad).

### Pilot study

Initially, face and content validation was conducted among a small targeted sample of randomly selected respondents meeting the inclusion criteria. All participants reported that the questionnaire items were simple, clear, and aligned with the study objectives. Content validity was evaluated to determine the degree to which the instrument sufficiently covered the construct of interest. Each item was reviewed by the aforementioned experts who rated the item's content validity in terms of its relevance. The internal consistency of the questionnaire was assessed using an alpha value of 0.785, indicative of adequate questionnaire reliability. Furthermore, sampling adequacy was assessed through Bartlett’s test of sphericity and the Kaiser–Mayer–Olkin (KMO) measure. The KMO value was 0.802, and Bartlett’s test of sphericity was significant (df = 435, *p* < 0.001), suggesting adequate sampling adequacy.

### Data collection and analysis

Questionnaires were distributed online via WhatsApp, emails, and Instagram over a 1-month period (March to April 2023). Participation was voluntary, and respondents could withdraw at any time by not completing the questionnaire or closing the questionnaire page. No participant-identifying information was collected.

## Results

### Respondents demographics

The respondents’ demographics are shown in Table 1. A total of 222 respondents completed the survey. The majority of them were female (*n* = 153, 68.9%) and of Brunei nationality (*n* = 190, 85.6%). The majority of the respondents were from the age group 20–29 years (*n* = 138, 62.2%). A higher proportion of respondents had an undergraduate degree (*n* = 90, 40.5%), and 85.1% (*n* = 189) completed their studies in Brunei. Most respondents are currently staying in the Brunei-Muara district (*n* = 177, 79.7%).

**Table 1:** Respondents demographics (*n* = 222)

Characteristics		Frequency, *n*	Percentage (%)
Gender	Male	69	31.1
	Female	153	68.9
Age (years)	18–19	20	9.0
	20–29	138	62.2
	30–39	37	16.7
	40–49	14	6.3
	50–59	7	3.2
	≥ 60	6	2.7
Nationality	Bruneian	190	85.6
	Permanent resident	22	9.9
	Others	10	4.5
Highest Education Level	Postgraduate	30	13.5
	Undergraduate	90	40.5
	Diploma	54	24.3
	A Levels	33	14.9
	O Levels	14	6.3
	Others	1	0.5
Country completed study	Brunei	189	85.1
	United Kingdom	14	6.3
	Australia	5	2.3
	United States of America	3	1.4
	Malaysia	3	1.4
	Others	8	3.6
District of residency	Brunei Muara	177	79.7
	Tutong	23	10.4
	Belait	18	8.1
	Temburong	4	1.8

### Pharmacy usage and preference for services

Table 2 presents the respondents' preferred location for medical treatment, willingness to use services and preferences, and frequency of visits to community pharmacies. Nearly equal proportions of respondents preferred to receive medical treatment in public/government institutions (48.6%, *n* = 108) and private clinics (43.7%, *n* = 97), while 7.7% preferred private pharmacies. When asked about the reason, the majority responded, ‘I am used to it’ (34.2%, *n* = 76). Cost was another factor mentioned by respondents (4.1%), with public and government facilities being cheaper or free of charge. Private clinics were favored for their shorter waiting times and better quality of medicines (1.4%), while private pharmacies were chosen for their convenience. Additional reasons provided (*n* = 9) include effective and accurate treatment, professionalism and experience of pharmacists, ease of obtaining medications, greater trust in government and available facilities.

**Table 2:** Preference for medical treatment, services, and visits to community pharmacies

Questions	Frequency, *n*	Percentage (%)
Preferred place to receive medical treatment		
Public/government	108	48.6
Private clinic	97	43.7
Private pharmacies	17	7.7
Why?		
My doctor asked me to	7	3.2
I am used to it	76	34.2
People around me visited there	11	5
Less waiting time	53	23.9
It is more convenient for me	54	24.3
Others	21	9.5
How often do you visit the community pharmacy?		
Once a week	3	1.4
Once a month	16	7.2
Once every few months	203	91.4
When was the last time you visited the pharmacy?		
A week ago	24	10.8
Few weeks ago	25	11.3
Last month	27	12.2
Few months ago	90	40.5
A year ago	16	7.2
More than a year ago	40	18
What is your purpose of visiting the community pharmacy?		
Collect medicines from prescription	104	46.8
Purchase over-the-counter medications	92	41.4
Counselling for medication uses	8	3.6
Ask for advice on medications	9	4.1
Others	9	4.1
Would you be willing to use other services provided by the pharmacy, other than the ones stated above?		
Yes	117	52.7
No	11	5
Maybe	94	42.3
If yes, what are the services you wish to receive from the community pharmacies? (*n* = 117)		
Screening services (e.g., blood pressure, blood glucose)	85	72.6
Treating minor illnesses (e.g., cough and cold, diarrhoea, constipation)	79	67.5
Skin care management	75	64.1
Supplements and herbal medicine recommendation/counselling	59	50.4
Medicine use review	53	45.3
Lifestyle advice	51	43.6
Vaccination	51	43.6
Follow-up services	48	41
Weight management program	47	40.2
Osteoporosis (bone problems)	29	24.8
Asthma care	26	22.2
Smoking cessation	15	12.8
Others	5	4.3

Regarding frequency of visits to community pharmacies, the majority of respondents (91.4%, *n* = 203) reported visiting once every few months, 7.2% (*n* = 16) once a month, and 1.4% (*n* = 3) once a week. Approximately 40.5% (*n* = 90) of respondents last visited a community pharmacy a few months ago. Close to half of the respondents (46.8%, *n* = 104) reported visiting community pharmacies to collect prescription medicines, while 41.4% (*n* = 92) visited to purchase over-the-counter (OTC) medications. Fewer respondents visit the pharmacy for medication advice (*n* = 9) and counselling for medication use (*n* = 8). Other reasons for visiting community pharmacies (*n* = 9) included having flu, fever, or cough, injuries, and seeking toiletries, with some reporting rarely visiting the community pharmacy.

Respondents were asked about their willingness to use services provided by community pharmacies, with answer options being ‘Yes’, ‘No’, and ‘Maybe’. Over half (52.7%) selected 'yes', 5% ‘no’, and 42.3% ‘maybe’. Respondents showed a high level of acceptance of services provided by community pharmacies, with all options being chosen at least once. Among respondents who selected ‘Yes’, ‘screening services’ (72.6%, 85/117), ‘treating minor illnesses’ (67.5%, 79/117), and ‘skin care management’ (64.1%, 75/117) were the most preferred services, while ‘smoking cessation’ (12.8%, 15/117) was the least preferred. Additional services mentioned include the availability of traditional medicines and reminding patients to take their medicines.

### Understanding of pharmacists and satisfaction with pharmacists

This section focused on the respondents’ understanding of the pharmacists’ role, their experience with the pharmacists as well as their satisfaction. The details of the responses are shown in Tables 3 and 4. Majority of respondents (*n* = 179, 80.6%) were aware of the role of a pharmacist, as shown in Table 3, ‘Dispense medicines’ (*n* = 194, 87.4%), ‘Counselling patients regarding their medications’ (*n* = 169, 76.1%), and ‘Collaborate with doctors in monitoring patient medication use’ (*n* = 126, 56.8%) were the most perceived role of a pharmacist, while ‘Policymakers’ (*n* = 17, 7.7%) and ‘Businessmen’ (*n* = 24, 10.8%) were the least perceived role.

**Table 3:** Perception of the role of pharmacists, experience with community pharmacists, preference for treatment of minor illnesses, and expectations of pharmacists

Questions	Frequency, *n*	Percentage (%)
Are you aware of the role of a pharmacist?		
Yes	179	80.6
No	43	19.4
What do you think pharmacists do?		
Dispense medicines	194	87.4
Counselling patients regarding their medications	169	76.1
Collaborate with doctors in monitoring patient medication use	126	56.8
Buying and selling medicines	108	48.6
Monitor patient medication therapy	81	36.5
Health promotion	72	32.4
Medication delivery service	63	28.4
Home medicine review	48	21.6
Businessmen	24	10.8
Policymakers	17	7.7
I do not know	1	0.5
All of the above	1	0.5
Do the pharmacists explain the use of the medicines, the possible side effects, and any warnings when dispensing?		
Yes	174	78.4
No	48	21.6
Have you ever asked pharmacists for advice regarding your medicine and condition?		
Yes	119	53.6
No	103	46.4
Do pharmacists provide counselling services?		
Yes	66	29.7
No	156	70.3
If your answer was yes to the previous question, please rate your satisfaction with the counselling services (*n* = 65)		
Very satisfied	7	10.8
Satisfied	25	38.5
Neutral	22	33.8
Unsatisfied	8	12.3
Very unsatisfied	3	4.6
Do the pharmacists answer your queries well?		
Yes	182	82
No	40	18
Where do you normally go when you have a minor illness (e.g., cough and cold, diarrhea, constipation)?		
To see a doctor in the hospital or private clinic	117	52.7
To purchase OTC medicines from the pharmacy nearby (e.g., Guardian)	91	41
Others	14	6.3
What are your expectations from a pharmacist		
Able to tell me about the medication (use, strength, dosage, how to take it)	201	90.5
Able to tell me about the possible side effects and potential warnings	197	88.7
Should ask me all relevant questions (medical conditions, medications, allergies etc.) before dispensing the medication	149	67.1
Able to tell me how to use my medical devices	148	66.7
Able to answer all my queries	141	63.5
Able to detect any medication error and prevent them from happening	141	63.5
Able to respect my confidentiality	136	61.3
Able to contact my doctor if there is anything they need to clarify for my prescription	127	57.2
Should be an expert in OTC medicines	102	45.9
Able to offer some lifestyle advice related to my condition	87	39.2
Others	5	2.3

**Table 4:** Satisfaction of pharmacies and pharmacists

Questions	Very satisfied *n* (%)	Satisfied *n* (%)	Neutral *n* (%)	Unsatisfied *n* (%)	Very Unsatisfied *n* (%)
Please rate your satisfaction of the pharmacy’s waiting time	15 (6.8)	48 (21.6)	107 (48.2)	39 (17.6)	13 (5.9)
Please rate your satisfaction of the pharmacists’ knowledge	23 (10.4)	77 (34.7)	98 (44.1)	20 (9.0)	4 (1.8)
Please rate your satisfaction of the pharmacists’ attitude	29 (13.1)	70 (31.5)	97 (43.7)	19 (8.6)	7 (3.2)
Please rate your satisfaction of the pharmacists’ professionalism	36 (16.2)	74 (33.3)	86 (38.7)	20 (9.0)	6 (2.7)
Please rate your satisfaction of the pharmacists communication skills	34 (15.3)	75 (33.8)	86 (38.7)	22 (9.9)	5 (2.3)

	Very good *n* (%)	Good *n* (%)	Neutral *n* (%)	Bad *n* (%)	Very bad *n* (%)
Please rate your overall impression of the pharmacists	23 (10.4)	75 (33.8)	106 (47.7)	15 (6.8)	3 (1.4)
Please rate your relationship with the pharmacists	14 (6.3)	53 (23.9)	140 (63.1)	9 (4.1)	6 (2.7)

Table 3 shows that 78.4% (*n* = 174) of respondents mentioned that the pharmacists explain the use of the medicines, the possible side effects, and any warnings when dispensing. More than half (*n* = 119, 53.6%) of the respondents had the experience of asking pharmacists for advice. It was also noted that most respondents (*n* = 156, 70.3%) were not offered counselling services by the pharmacists. Among those that have received counselling, they had positive views on the counselling sessions as several respondents were very satisfied (*n* = 7, 10.8%) or satisfied (*n* = 25, 38.5%) with the sessions. In addition, 82.0% (*n* = 182) of respondents reported that the pharmacists answered their queries well.

When asked about where they would go when they have minor illnesses, 52.7% (*n* = 117) of respondents chose to see a doctor in the hospital or private clinic and 41.0% (*n* = 91) to purchase OTC medicines from the pharmacy nearby. 3.6% (*n* = 8) of respondents would prefer to stay at home and rest until their condition improves (Table 3).

Respondents were then asked about their expectations of a pharmacist. All options were chosen at least once, with ‘Able to tell me about the medication (use, strength, dose, how to take it), and ‘Able to tell me about the possible side effects and potential warnings’ being the most selected options, at 90.5% (*n* = 201) and 88.7% (*n* = 197), respectively. Respondents least expect pharmacists to offer lifestyle advice related to their condition, with 39.2% (*n* = 87) of respondents choosing it. Other answers provided include being polite when talking to a patient over the counter as well as good customer service. Details of the respondents’ expectations are summarized in Table 3.

The following questions required respondents to rate their satisfaction with several aspects of the pharmacists and pharmacy on a scale of 1–5, with 1 being very satisfied/very good and 5 being very unsatisfied/very bad. Overall, all factors were positively viewed by most respondents, although the percentage of respondents who had positive views on the pharmacy’s waiting time was similar to that of the negative view; 28.4% (*n* = 63) vs 23.5% (*n* = 52), respectively (Table 4).

### Views on the dispensing separation system

This section explores the respondents’ views on the concept of dispensing separation (DS), a system, where the role of prescribing and dispensing medications is separated, usually performed by different healthcare professionals.

A significant majority of respondents were not familiar with the term “dispensing separation” (76.1%, *n* = 169), and many were unaware of its implementation in other countries (84.7%, *n* = 188). When asked if Brunei should implement this system, most respondents expressed uncertainty (57.7%, *n* = 128), with a similar proportion also unsure whether Brunei is prepared for such a system (62.6%, *n* = 139).

However, a notable number of respondents did believe that Brunei should implement the DS system (36.5%, *n* = 81) and felt prepared for its execution (24.8%, *n* = 55). Those who supported the DS system’s implementation cited reasons, such as improved safety, convenience, and adequate manpower. Conversely, respondents who disagreed with its implementation mainly pointed to a perceived lack of manpower and a preference for the existing practice.

Among those unsure about whether Brunei is ready for DS system implementation, the most common reason given was unfamiliarity with the DS system and a lack of knowledge about it. The details of respondents’ views on DS are summarized in Table 5.

**Table 5:** Respondents’ views on dispensing separation

Questions	Frequency, *n*	Percentage (%)
Have you heard of the term dispensing separation?		
Yes	24	10.8
No	169	76.1
Maybe	29	13.1
Are you aware that other countries have implemented the dispensing separation system? (e.g., Japan, Korea, Taiwan)		
Yes	34	15.3
No	188	84.7
Do you think Brunei should implement the dispensing separation system? (For the private sector)		
Yes	81	36.5
No	13	5.9
I do not know	128	57.7
Do you think Brunei is prepared to implement the dispensing separation system? (For the private sector)		
Yes	55	24.8
No	28	12.6
I do not know	139	62.6
Why do you think so?		
Not familiar with the DS system and lack of information about it	111	72.5
For Safety	12	7.8
Convenience	7	4.6
Enough staff and manpower	5	3.3
To improve or move forward	4	2.6
Brunei has the resource to implement the system	4	2.6
It is time	3	2.0
Reduce waiting time	3	2.0
Efficient	2	1.3
If it is practiced in public, then private can too	2	1.3

### Private community pharmacies

A significant number of respondents reported that there is always a pharmacist available (*n* = 81, 36.5%) and that pharmacists or staff asked for relevant medical histories before selling medication (*n* = 77, 34.7%). However, many respondents are unaware of the services provided by private community pharmacies (*n* = 154, 69.4%), and a considerable proportion have never or rarely been to a community pharmacy. Details of the responses are shown in Table 6.

**Table 6:** Experience in private community pharmacies

Questions	Yes *n* (%)	No *n* (%)	I have never or rarely been to a private community pharmacy *n* (%)
There is always a pharmacist available	81 (36.5)	32 (14.4)	109 (49.1)
The pharmacists or staff asked me about my medication history, medical condition history, allergies, and symptoms before selling the medication	77 (34.7)	45 (20.3)	100 (45.0)
Are you aware of any services offered by private community pharmacies?	68 (30.6)	154 (69.4)	–
The pharmacist or staff tells me about the use, dosage, possible side effects, warnings, and other relevant information about the medicine before selling/dispensing it to me	98 (44.1)	26 (11.7)	98 (44.1)
The pharmacist or staff answer my queries well	108 (48.6)	15 (6.8)	99 (44.6)
The pharmacist spends enough time with me	85 (38.3)	32 (14.4)	105 (47.3)

### Understanding of P medicines

More than half of the respondents (*n* = 128, 58.1%) do not know what a P medicine is. Among those who have ever purchased a P medicine, the majority (*n* = 53, 79.1%) bought it from private community pharmacies. Details are summarized in Table 7. The respondents were almost equally divided on whether Brunei should classify more medicines as P medicines, so that consumers can buy them at community pharmacies, with slightly more respondents (50.9%) being unsure, as compared to respondents that agree (41.9%).

**Table 7:** Knowledge of P medicines

Questions	Frequency,* n*	Percentage (%)
Do you know what P medicine is?		
Yes	93	41.9
No	129	58.1
Have you ever purchased any P medicine?		
Yes	67	30.2
No	67	30.2
Not sure	88	39.6
If yes, where do you buy it from?		
Hospital	12	17.9
Private community pharmacies	53	79.1
Others	2	3.0
Do you think Brunei should classify more medicines as P medicines, so that consumers can buy them at community pharmacies?		
Yes	93	41.9
No	16	7.2
No comment	113	50.9

### Relationship between demographic variables, satisfaction with pharmacists, views on dispensing Separation, private community pharmacies, and P medicines

One-way ANOVA and independent *t* test were used for comparison. No significant differences were found between demographic variables and their satisfaction with the pharmacists (see Appendix 2).

However, significant differences were found among different age groups regarding the view score on dispensing separation system (*p* = 0.021), as shown in Table 8. The participants of age 41–50 years were having high score, as shown in Table 8.

**Table 8:** Views score on dispensing separation system vs private community pharmacies and satisfaction score having/with pharmacist

Characteristics		View score on dispensing separation system		Satisfaction score having/with pharmacist	
		Mean ± SD	*p* value	Mean ± SD	*p* value
Gender	Male	3.25 ± 2.58	0.723 a	17.66 ± 5.10	0.356 a
	Female	3.09 ± 2.55		16.90 ± 5.10	
Age (years)	18–30	3.04 ± 2.42	0.021 b*	17.04 ± 4.93	0.926 b
	31–40	3.94 ± 2.85		17.21 ± 4.37	
	41–50	4.08 ± 2.72		17.07 ± 3.88	
	51–60	3.88 ± 3.40		18.37 ± 2.26	
	≥ 60	0.40 ± 0.89		18.20 ± 1.48	
Highest Education Level	Postgraduate	3.24 ± 2.55	0.891 b	16.88 ± 4.65	0.055 b
	Undergraduate	3.00 ± 2.71		19.40 ± 3.88	
	Diploma	3.00 ± 2.75		17.10 ± 5.36	
District of residency	Brunei Muara	3.29 ± 2.61	0.569 b	16.71 ± 4.62	0.052 b
	Tutong	2.61 ± 2.17		18.60 ± 4.46	
	Belait	3.33 ± 2.74		19.00 ± 4.93	
	Temburong	2.25 ± 1.89		19.50 ± 1.73	

a = Independent *t* test; b = One-way ANOVA; *p value less than 0.05 statistically significant

Binary logistic regression was used for the comparison of related to understanding of P medicines; however, no statistically significant difference was observed among the response in comparison with gender, age, education, and district of residence (see Appendix 2).

## Discussion

This study evaluated the perceptions of the general public towards the services offered by community pharmacies, their interactions with and comprehension of community pharmacists, opinions on dispensing separation, private community pharmacies, and understanding of P medicines.

### Pharmacy usage and preference for services

The respondents indicated a preference for receiving medical treatment from public institutions, government organizations, or private clinics, reflecting a potentially stronger trust in physicians [19, 20]. A study examining consumer perceptions of community pharmacists revealed a predilection for physicians in the context of health problem consultations [19].

Most respondents favoring public institutions or private clinics attributed this preference to familiarity or convenience. The shorter wait times at private clinics were highlighted as a distinct advantage. Furthermore, respondents who preferred public institutions often cited financial considerations, as services in Brunei’s public institutions are typically free or charged at a lower rate compared to private clinics and pharmacies, with just a nominal $1 registration fee.

The majority of respondents reported visiting community pharmacies every few months primarily to collect prescribed medications or buy over-the-counter drugs, mirroring findings from studies conducted in other countries [18, 19, 21]. This underlines a globally common perception of the principal roles of community pharmacies.

Specialized services, such as asthma and hypertension management, along with osteoporosis screening, were demonstrated to improve patients’ conditions and clinical outcomes (e.g., reduced blood pressure and increased peak expiratory flow) [22]. Screening programs also proved effective in enhancing disease detection and improving referral rates [23].

In our study, respondents displayed a readiness to utilize extended services if offered by community pharmacies. Screening services and treatment of minor illnesses received significant interest, whereas smoking cessation was least favored. A study conducted in Palestine found similar willingness among respondents to use extended services provided by community pharmacies [19]. It revealed a high consumer demand for such services, especially for screening procedures (blood cholesterol, blood glucose, blood pressure monitoring) and measurements of weight, height, and temperature [19]. A study from England indicated that participants were highly receptive to services, particularly health checks and advice related to cardiac conditions. Minor illnesses, being of low risk, are often deemed suitable for pharmacist-led treatment, while GP visits are reserved for chronic conditions [20].

A study from England also noted that smoking cessation services and alcohol advice were less popular [24]. One plausible explanation for the low popularity of smoking cessation in our study is the possibility of the respondents being non-smokers, therefore, having no interest in the service. Intriguingly, several respondents expressed interest in receiving medication reminders and appreciated the provision of traditional medicines.

### Understanding of and satisfaction with pharmacists

The majority of respondents were cognizant of a pharmacist’s traditional role, primarily dispensing medicines (87.4%). In addition, counseling patients about their medications (76.1%) and collaborating with doctors to monitor patient medication (56.8%) were also identified as common roles of pharmacists. These findings align with a study by Mukattash et al., wherein dispensing medicines (46.2%) and counseling patients (34.6%) were recognized as the most crucial tasks of a pharmacist [14].

The study results indicated that respondents were well-versed with medication use, potency, and all relevant information concerning the medication before it was dispensed. Similarly, El-Kholy et al. reported that 72.8% of respondents concurred that pharmacists provided clear instructions on medication use [18].

Counseling services aim to enhance patients' understanding of their medicines and promote adherence [25]. Despite a significant proportion of respondents recognizing medication counseling as a typical role of a pharmacist, most respondents had not received these services. Potential barriers to providing counseling services could include time constraints or excessive workload of the pharmacists [6]. Among respondents who had received counseling, most expressed satisfaction with the session, appreciating the pharmacist’s knowledge and communication skills.

Respondents preferred to consult doctors in public institutions or private clinics for minor illnesses, consistent with their preferred destination for general medical treatment. However, the number of respondents choosing private pharmacies increased, suggesting that some trust private pharmacies to treat minor illnesses [19]. Gidman et al. reported that participants were more likely to visit community pharmacists for conditions deemed ‘low risk’ [20]. Some respondents might also be managing their minor conditions independently, viewing a doctor's visit as unnecessary [26].

Respondents demonstrated high expectations of pharmacists. Nearly all expected pharmacists to provide relevant information (use, strength, dosage, instructions for use, potential side effects, and warnings) about the medication before dispensing. This suggests a high degree of trust in the pharmacists' knowledge of medication. Another study reported that pharmacists were expected to counsel about potential interactions with other medications, use of the medications, and disease-related counseling [27]. Pharmacists are expected to provide drug information when dispensing medications, enabling patients to better understand their medication, be aware of potential side effects, and improve adherence [24]. Respondents also expected pharmacists to inquire about medical and medication history before dispensing and provide education on the use of their medical devices.

In general, respondents expressed satisfaction with the pharmacist's knowledge, attitude, professionalism, and communication skills, mirroring findings from a study that analyzed online patient feedback about pharmacies, where staff attitudes were perceived positively [28]. The impression of and relationship with the pharmacist were also viewed favorably.

While the general sentiment remained positive, a slightly higher proportion of respondents expressed dissatisfaction with the pharmacy’s waiting time. Similar findings have been reported in other studies [24, 28], with waiting times to receive medications from community pharmacies perceived as excessively long, ranging from hours to days [28]. An acceptable waiting time, not exceeding 15 min, was reported by respondents in an English study [24]. In addition, extended waiting times have been associated with decreased satisfaction [18].

### Views on the dispensing separation system

The dispensing separation (DS) practice, where doctors solely prescribe and pharmacists dispense, is utilized in other countries and the public sector in Brunei. This system aims to enhance patient safety and reduce medication errors by enabling pharmacists to double-check prescriptions and prevent overprescribing [29].

The majority of respondents were unfamiliar with the term dispensing separation or its implementation overseas. Results from Malaysian studies revealed that 65% of respondents from Malaysia were unaware of DS [29], while 67.5% knew about the DS system implemented in other countries [15].

### Private community pharmacies

Nearly half of the respondents had never visited private community pharmacies, possibly because there are fewer such establishments in Brunei, and they are not as heavily utilized as government community pharmacies located in health centres.

Among respondents who had visited private community pharmacies, most reported positive experiences, indicating the presence of a pharmacist, the gathering of relevant medical history, and the provision of medication information during the dispensing process. These results align with a study by El-Kholy et al., in which a majority of respondents confirmed the presence of a pharmacist to assist them (74.6%) and acknowledged that the pharmacist asked about their medication (45.5%) and medical conditions (49.1%) when preparing the prescription [18].

Despite visiting community pharmacies, respondents were largely unaware of the services provided there. This suggests that pharmacy owners or pharmacists may not be effectively promoting their services to their customers.

Respondents also expressed satisfaction with the pharmacist’s knowledge and communication skills, and felt that their queries were well-addressed. Pharmacists are often viewed as medication experts, and the public generally prefers consulting them for medication inquiries over doctors [14, 29]. Mukattash et al. found that the majority of respondents prefer to acquire information about medication from pharmacists [14]. However, a study in Qatar revealed that only 37% of respondents felt community pharmacists were knowledgeable enough and answered their questions satisfactorily, which is a lower percentage than found in this study (48.6%) [30].

In addition, respondents expressed satisfaction with the attention and time dedicated to them, suggesting that pharmacists devote substantial time to each customer to ensure adequate care [14].

In summary, the results indicate a satisfactory level of contentment with the availability and practices of pharmacists. However, gaps persist in public knowledge and usage of pharmacy services. These gaps could potentially be addressed through educational initiatives and awareness campaigns aimed at improving public comprehension of these issues.

The association between demographic variables and respondents’ views implies that specific strategies might need to be tailored to different age and gender groups for effective communication and implementation of changes in Brunei’s pharmacy sector.

### Understanding of P medicines

Pharmacy (P) medicines can be purchased without a prescription in the presence of a pharmacist [16]. They are usually kept behind the counter and not displayed to the public.

The study’s results revealed that respondents are largely unfamiliar with the classification of medicines, as many did not know what a P medicine is and were unsure if they had ever bought one before. Among those who had bought P medicines before, a higher proportion had purchased them from private community pharmacies.

When asked whether Brunei should classify more medicines as P medicines to increase their availability at community pharmacies, respondents neither agreed nor disagreed strongly. This could be due to respondents’ lack of knowledge about P medicines and the potential benefits and risks associated with reclassification. Consequently, if Brunei intends to expand the P medicine category, it is important to assess both public understanding and pharmacists’ competency before making changes. The public should have adequate understanding of their conditions, while pharmacists should possess enough knowledge of the conditions and medications to provide appropriate recommendations.

The study’s results indicate a significant lack of awareness about key aspects of pharmaceutical services among respondents in Brunei. This includes unfamiliarity with the concept and implementation of a dispensing separation system, services provided by community pharmacies, and the classification of P medicines.

#### Limitations

The primary limitation of this study was the time constraints. Due to these constraints and the low number of respondents, the target sample size was not met. In addition, the majority of respondents were female, potentially skewing results towards the perspectives of the female population over the male population. Moreover, the study employed online questionnaires, which may have limited the participation of older generations. The respondents were also largely from an undergraduate educational background, but the study did not probe further into their specific fields of study. This limited the study's ability to correlate educational field with respondents’ understanding and in-depth knowledge, posing another potential limitation.

### Recommendations for future research

To address the limitations of this study, several recommendations can be made for future research. First, efforts should be made to recruit an equal proportion of male and female respondents to avoid a skewed gender ratio. Second, in-person surveys could be conducted to reach a wider and more diverse demographic, including older populations. Furthermore, future research could explore the perceptions of healthcare professionals and stakeholders regarding the feasibility of dispensing separation (DS) in Brunei through qualitative studies.

## Conclusion

In general, the public in Brunei primarily prefers to visit doctors for medical treatment and uses community pharmacies predominantly for prescription collection or purchasing over-the-counter medications. Nevertheless, they are open to using additional services provided by community pharmacists, particularly screening services and treatment for minor illnesses. However, public knowledge about the dispensing separation system and the classification of medications is limited. As such, educational and awareness initiatives will be crucial in enhancing public understanding of pharmaceutical services and the roles of pharmacists before implementing any changes in the sector.

## Appendix 1

### Section 1: demographics

1. **Gender**
2. Female
3. Male
4. **Age (years)**
5. **Nationality**
6. Bruneian
7. Permanent resident
8. Other:
9. **Highest education level**
10. Postgraduate
11. Undergraduate
12. Diploma
13. Sixth form graduate (A Levels)
14. Secondary graduate (O Levels)
15. Others
16. **Please state the country that you have completed your undergraduate and graduate study**
17. **Which district do you live in**
18. Brunei Muara
19. Tutong
20. Belait
21. Temburong

### Section 2: pharmacy usage and preference of services

7. **Please state your preferred place to receive usual medical treatment**
8. Public/government institution
9. Private clinic
10. Private pharmacy (such as Guardian Pharmacy)
11. **Why?**
12. My doctor asked me to
13. I am used to it
14. People around me visited there
15. Less waiting time
16. It is more convenient for me
17. Other
18. **How often do you visit the community pharmacy?**
19. Once a week
20. Once a month
21. Once every few months
22. Other
23. **When was the last time you visited the pharmacy?**
24. A week ago
25. Few weeks ago
26. Last month
27. Few months ago
28. A year ago
29. More than a year ago
30. **What is your purpose of visiting the community pharmacy?**
31. Collect medicines from prescription
32. Purchase over-the-counter medications
33. Counselling for medication uses
34. Ask for advice on medications
35. Other
36. **Would you be willing to use other services provided by the pharmacy, other than the ones stated above?**
37. Yes
38. No
39. Maybe
40. **If yes, what are the services you wish to receive from the community pharmacies?**
41. Screening services (e.g., blood pressure, blood glucose)
42. Medicine use review
43. Smoking cessation
44. Lifestyle advices
45. Weight management program
46. Vaccination
47. Asthma care
48. Supplements and herbal medicine recommendation/counselling
49. Skin care management
50. Osteoporosis care (bone problems)
51. Treating minor illness (e.g., cough and cold, diarrhea, constipation)
52. Follow-up services
53. Other

### Section 3: understanding of pharmacists and satisfaction with pharmacists

14. **Are you aware of the role of a pharmacist?**
15. Yes
16. No
17. **What do you think pharmacists do? (Tick all that applies)**
18. Dispense medicines
19. Counselling patients regarding their medications
20. Buying and selling medicines
21. Monitor patient medication therapy
22. Health promotion
23. Collaborate with doctors in monitoring patient medication use
24. Medication delivery service
25. Home medicine review
26. Policymakers
27. Businessmen
28. Other
29. **Do the pharmacists explain the use of the medicines, the possible side effects, and any warnings when dispensing?**
30. Yes
31. No
32. **Have you ever asked the pharmacists for advice regarding your medicine and condition?**
33. Yes
34. No
35. **Do the pharmacists provide counselling services?**
36. Yes
37. No
38. **If your answer was yes to the previous question, please rate your satisfaction of the counselling services**
39. 1 (very satisfied) to 5 (very unsatisfied)
40. **Do the pharmacists answer your queries well?**
41. Yes
42. No
43. **Where do you normally go when you have a minor illness (e.g., cough and cold, diarrhea, constipation)?**
44. To see a doctor in the hospital or private clinic
45. To purchase OTC medicines from the pharmacy nearby (e.g., Guardian)
46. Other
47. **What are your expectations of a pharmacist? (Tick all that applies)**
48. Able to tell me about the medication (use, strength, dosage, how to take it)
49. Able to tell me about the possible side effects and potential warnings
50. Should be an expert in OTC medicines
51. Able to tell me how to use my medical devices
52. Should ask me all relevant questions (medical conditions, medications, allergies etc.) before dispensing the medication
53. Able to answer all my queries
54. Able to offer some lifestyle advices related to my condition
55. Able to respect my confidentiality
56. Able to contact my doctor if there is anything they need to clarify for my prescription
57. Able to detect any medication error and prevent them from happening
58. Other
59. **Please rate your satisfaction of the pharmacy’s waiting time**
60. 1 (very satisfied) to 5 (very unsatisfied)
61. **Please rate your overall impression of the pharmacists**
62. 1 (very good) to 5 (very bad)
63. **Please rate your satisfaction of the pharmacists’ knowledge**
64. 1 (very satisfied) to 5 (very unsatisfied)
65. **Please rate your satisfaction of the pharmacists’ attitude**
66. 1 (very satisfied) to 5 (very unsatisfied)
67. **Please rate your satisfaction of the pharmacists’ professionalism**
68. 1 (very satisfied) to 5 (very unsatisfied)
69. **Please rate your satisfaction of the pharmacists’ communication skills**
70. 1 (very satisfied) to 5 (very unsatisfied)
71. **Please rate your relationship with the pharmacists**
72. 1 (very good) to 5 (very bad)

### Section 4: views on the dispensing separation system

Dispensing separation is the separation between prescribing and dispensing, where physicians solely prescribe and pharmacists solely dispense. In Brunei, it is practiced in the hospitals and government health centres but not in private clinics and pharmacies. In the private sector, the doctor will both prescribe and dispense or the dispensing will normally be done by a non-pharmacist.

30. **Have you heard of the term dispensing separation?**
31. Yes
32. No
33. Maybe
34. **Are you aware that other countries have implemented the dispensing separation system? (e.g., Japan, Korea, Taiwan)**
35. Yes
36. No
37. **Do you think Brunei should implement the dispensing separation system? (For private sector)**
38. Yes
39. No
40. I do not know
41. **Do you think Brunei is prepared to implement the dispensing separation system? (For private sector)**
42. Yes
43. No
44. I do not know
45. **Why do you think so?**

### Section 5: private community pharmacies

35. **There is always a pharmacist available**
36. Yes
37. No
38. I have never or rarely been to a private community pharmacy
39. **The pharmacists or staff asked me about my medication history, medical condition history, allergies, symptoms before selling the medication**
40. Yes
41. No
42. I have never or rarely been to a private community pharmacy
43. **Are you aware of any services offered by the private community pharmacies?**
44. Yes
45. No
46. **The pharmacist or staff tells me about the use, dosage, possible side effects, warnings, and other relevant information of the medicine before selling/dispensing it to me**
47. Yes
48. No
49. I have never or rarely been to a private community pharmacy
50. **The pharmacist or staff answer my queries well**
51. Yes
52. No
53. I have never or rarely been to a private community pharmacy
54. **The pharmacist spends enough time with me**
55. Yes
56. No
57. I have never or rarely been to a private community pharmacy

### Section 6: understanding of P medicines

Pharmacy (P) medicines are medicines that can only be bought from a pharmacy and in the presence of a pharmacist. They are normally stored behind the counter in pharmacies and are not in the open areas.

41. **Do you know what a P medicine is?**
42. Yes
43. No
44. **Have you ever purchased any P medicine?**
45. Yes
46. No
47. Not sure
48. **If yes, where do you buy it from?**
49. Hospital
50. Private community pharmacies
51. Other
52. **Do you think Brunei should classify more medicines as P medicines, so that consumers can buy them at community pharmacies**
53. Yes
54. No
55. No comment

## Appendix 2 Comparison related to the understanding of P medicines

**Table:** 

	Do you know what P medicine is?	Have you ever purchased any P medicine?	Do you think Brunei should classify more medicines as P medicines, so that consumers can buy them at community pharmacies?
	OR (95%CI)	OR (95%CI)	OR (95%CI)
Gender			
Male	Reference	Reference	Reference
Female	1.540 (0.855;2.775)	1.335 (0.707;2.251)	1.288 (0.720;2.307)
Age in years			
18–30 years	Reference	Reference	Reference
31–40 years	0.996 (0.467; 2.124)	0.709 (0.323; 1.556)	0.947 (0.444; 2.020)
41–50 years	0.629 (0.202; 1.955)	0.473 (0.151; 1.481)	0.310 (0.092; 1.049)
51–60 years	1.223 (0.283; 5.293)	1.216 (0.237; 6.240)	1.163 (0.269; 5.034)
More than 60 years			
Education			
Postgraduate	Reference	Reference	Reference
Undergraduate	1.051 (0.428; 2.5770	0.558 (0.238; 1.456)	0.556 (0.229; 1.350)
Diploma	1.091 (0.298; 3.993)	0.951 (0.237; 3.810)	0.667 (0.187; 2.381)
District of residency			
Brunei Muara	Reference	Reference	Reference
Tutong	1.379 (0.556; 3.420)	0.626 (0.478; 3.416)	1.067 (0.438; 2.596)
Belait	1.155 (0.428; 3.120)	0.773 (0.398; 3.450)	0.549 (0.207; 1.457)
Temburong	0.245 (0.025; 2.403)	0.796 (0.138; 13.295)	0.686 (0.094; 4.980)

Binary logistic regression was used, OR: Odd Ratio, CI: Confidence Interval; * *p* value less than 0.05 statistically significant.

## Data Availability

Please contact author for data requests.

## Abbreviations

DS: Dispensing separation
GDP: Good dispensing practice
CPSA: Community Pharmacy Services Agreement
POM: Prescription only medicines
P-medicine: Pharmacy medicine
GSL: General sales list
BDMCA: Brunei Darussalam Medicines Control Authority
UBD: Universiti Brunei Darussalam
UTB: Universiti Teknologi Brunei
UNISSA: Universiti Islam Sultan Sharif Ali
IBTE: Institute of Brunei Technical Education
LCB: Laksamana College of Business
PB: Politeknik Brunei
PAPRSB IHSREC: Pengiran Anak Puteri Rashidah Institute of Health Sciences Research Ethics Committee
